# Pharmacologic Therapies for Patent Ductus Arteriosus in Extremely Preterm Infants

**DOI:** 10.1001/jamanetworkopen.2026.17477

**Published:** 2026-06-09

**Authors:** Souvik Mitra, Amish Jain, Joseph Y. Ting, Nadya Ben Fadel, Christine Drolet, Ayman Abou Mehrem, Amuchou Singh Soraisham, Bonny Jasani, Deepak Louis, Anie Lapointe, Jon Dorling, Faiza Khurshid, Abbas Hyderi, Kumar Kumaran, Jennifer Toye, Andrei Harabor, Dany E. Weisz, Miroslav Stavel, Alyssa Morin, Soume Bhattacharya, Renjini Lalitha, Jehier Afifi, Sajit Augustine, Michael P. Castaldo, Tara Hatfield, Yi-Chen Su, Prakesh S. Shah

**Affiliations:** 1 Division of Neonatology, Department of Pediatrics, University of British Columbia, Vancouver, British Columbia, Canada; 2 Department of Pediatrics, Mount Sinai Hospital and University of Toronto, Toronto Ontario, Canada; 3 Department of Pediatrics, University of Alberta, Edmonton, Alberta, Canada; 4 Division of Neonatology, Department of Pediatrics, University of Ottawa, Ottawa, Ontario, Canada; 5 Laval University, Quebec City, Quebec, Canada; 6 Section of Newborn Critical Care, Department of Pediatrics, University of Calgary, Calgary, Alberta, Canada; 7 Section of Neonatology, Department of Pediatrics and Child Health, University of Manitoba, Winnipeg, Manitoba, Canada; 8 Division of Neonatology, Department of Pediatrics, University of Montreal, Montreal Quebec, Canada; 9 Child Health Outcomes Research at Leeds, University of Leeds, West Yorkshire, United Kingdom; 10 Department of Pediatrics, Queen’s University, Kingston, Ontario, Canada; 11 University of Saskatchewan, Saskatoon, Saskatchewan, Canada; 12 Centre Hospitalier Universitaire (CHU) Sherbrooke, Sherbrooke, Quebec, Canada; 13 Division of Neonatal-Perinatal Medicine, Western University, London, Ontario, Canada; 14 Division of Neonatal-Perinatal Medicine, Department of Pediatrics, Dalhousie University, Halifax, Nova Scotia, Canada; 15 Division of Neonatal-Perinatal Medicine, IWK Health, Halifax, Nova Scotia, Canada; 16 Royal Columbian Hospital, Fraser Health, New Westminster British Columbia, Canada; 17 Department of Pediatrics, The Hospital for Sick Children, Toronto, Ontario, Canada; 18 Newborn and Developmental Paediatrics, Sunnybrook Health Sciences Centre, Toronto, Ontario, Canada

## Abstract

**Importance:**

Wide variation exists in patent ductus arteriosus (PDA) treatment practices in extremely preterm infants (defined in this study as born before 29 weeks’ gestation). Commonly used treatment strategies include use of standard-dose and adjustable-dose ibuprofen, indomethacin, and acetaminophen as well as nonpharmacological conservative management.

**Objectives:**

To compare the relative effectiveness of different PDA pharmacotherapeutic regimens in extremely preterm infants, and to compare the clinical outcomes between infants who were treated with pharmacotherapy and those who received no pharmacotherapy (conservatively managed).

**Design, Setting, and Participants:**

This comparative effectiveness research study using clinical data from 19 tertiary neonatal intensive care units (NICUs) in Canada, which are part of the Canadian Neonatal Network, was conducted between January 1, 2020, and July 31, 2023. Infants born before 29 weeks’ gestation with echocardiography-confirmed, predominantly left-right shunting, moderate- to large-sized (diameter: ≥1.5 mm) PDA were included. Infants who received PDA treatment based solely on clinical diagnosis without echocardiographic confirmation or underwent primary procedural closure were excluded. Prior to study initiation, each NICU self-selected 1 of the following 4 predefined interventions as their primary pharmacotherapy regimen: standard-dose ibuprofen, adjustable-dose ibuprofen, indomethacin, and acetaminophen. Data analyses were conducted from May 9, 2024, to February 23, 2026.

**Exposure:**

Primary PDA pharmacotherapy using 1 of the following regimens: standard-dose ibuprofen, adjustable-dose ibuprofen, indomethacin, and acetaminophen.

**Main Outcomes and Measures:**

The primary outcome was failure of primary pharmacotherapy, defined as the need for additional medical and/or procedural PDA treatment. The secondary outcomes included repeat pharmacotherapy course, surgical or interventional PDA closure, predischarge mortality, moderate to severe bronchopulmonary dysplasia (BPD), stage 2 or higher necrotizing enterocolitis (NEC), and definite sepsis.

**Results:**

A total of 1356 infants (mean [SD] gestational age [GA], 25.4 [1.6] weeks; mean [SD] birth weight, 828 [228] g; 746 males [55.0%]) were included, of whom 1097 (80.9%) received PDA pharmacotherapy and 259 (19.1%) did not. Among the participating NICUs, 7 selected standard-dose ibuprofen, 8 selected adjustable-dose ibuprofen, 1 selected indomethacin, and 3 selected acetaminophen as their primary pharmacotherapy. There was 28.1% (308 of 1097) protocol deviation, 75.0% of which was attributable to the use of acetaminophen by sites that did not intend to use acetaminophen. Among infants who received treatment, 464 (42.3%) experienced failure of the primary pharmacotherapy, and 456 (41.6%) received repeat pharmacotherapy course. There were no differences between the 4 treatment regimens in failure of primary pharmacotherapy after adjustment for confounders and accounting for clustering within each site. Compared with infants who received no pharmacotherapy, treated infants had higher odds of moderate to severe BPD (adjusted odds ratio [AOR], 1.91; 95% CI, 1.12-3.25) and NEC (AOR, 2.15; 95% CI, 1.55-2.99) but lower odds of mortality (AOR, 0.35; 95% CI, 0.21-0.58).

**Conclusions and Relevance:**

In this comparative effectiveness research study, the rate of PDA pharmacotherapy failure was high among extremely preterm infants, with no observed differences in effectiveness between commonly used pharmacotherapy regimens. Compared with conservative management, pharmacotherapy was associated with lower mortality and higher BPD and NEC odds; however, confounding by contraindication and survival bias cannot be ruled out.

## Key Points

**Question:**

What is the relative effectiveness of different pharmacotherapeutic regimens for patent ductus arteriosus (PDA) in extremely preterm infants?

**Findings:**

In this comparative effectiveness study of 1356 extremely preterm infants (born before 29 weeks’ gestation), the overall primary PDA pharmacotherapy failure rate was 42.3%. There were no differences between 4 treatment regimens in failure of primary pharmacotherapy or need for repeat treatment, after adjustment for confounders and accounting for clustering within each participating neonatal intensive care unit.

**Meaning:**

In this study, no observed differences in effectiveness of primary PDA pharmacotherapy were found in extremely preterm infants regardless of medication choice.

## Teaser

This comparative effectiveness research study examines the failure rate and other clinical outcomes associated with use of standard-dose ibuprofen, adjustable-dose ibuprofen, indomethacin, and acetaminophen for treatment of patent ductus arteriosus among infants born before 29 weeks’ gestation.

## Introduction

The most common cardiovascular problem of extremely preterm infants is patent ductus arteriosus (PDA).^1^ Current pharmacological options for PDA include cyclooxygenase (COX) inhibitors such as indomethacin, ibuprofen, and acetaminophen. Based on earlier randomized clinical trials (RCTs), indomethacin was considered to be the gold standard.^1,2,3^ Subsequent RCTs suggested that ibuprofen was equally effective in closing a PDA as reducing adverse effects.^4^ The ibuprofen dose used in these RCTs was consistent and therefore referred to as standard-dose ibuprofen (10 mg/kg followed by 2 doses of 5 mg/kg at 24-hour intervals).^4^ Based on these studies, standard-dose ibuprofen was widely adopted as the first treatment choice for PDA.

However, the applicability of these trial results to routine clinical practice remains controversial.^5^ A bayesian network meta-analysis showed that higher ibuprofen doses may improve PDA closure.^6^ However, the effectiveness and safety of high-dose ibuprofen in extremely preterm infants are largely unknown, precluding its adoption. The controversy is evident from an unpublished 2019 survey conducted through the Canadian Neonatal Network (CNN) that identified 56% of responding centers used standard-dose ibuprofen while 32% used adjustable-dose ibuprofen.

Based on this practice variation, we aimed to prospectively compare the relative effectiveness of different PDA pharmacotherapeutic regimens in extremely preterm infants (defined in this study as born before 29 weeks’ gestation) and to compare the clinical outcomes between these infants who were treated with pharmacotherapy and those who received no pharmacotherapy (conservatively managed). We hypothesized that adjustable-dose ibuprofen would be associated with lower primary pharmacotherapy failure and that infants who received medical treatment for a moderate- to large-sized PDA would have improved clinical outcomes compared with infants who did not receive medical treatment.

## Methods

### Study Design

We conducted a multicenter, prospective, observational comparative effectiveness research study (ClinicalTrials.gov Identifier: NCT04347720) at 19 tertiary neonatal intensive care units (NICUs) between January 1, 2020, and July 31, 2023. The institutional review boards of the participating sites approved the study and waived the informed consent requirement because all sites are part of the CNN and anonymized data from the patient registry were used. We followed the International Society for Pharmacoeconomics and Outcomes Research (ISPOR) reporting guideline for comparative effectiveness research.^7^

### Participants and Interventions

Infants born before 29 weeks’ gestation with an echocardiography-confirmed, predominantly left-right shunting, moderate- to large-sized (diameter: ≥1.5 mm) PDA were included. Infants who received prophylactic indomethacin within the first 24 hours for intraventricular hemorrhage (IVH) prevention were also included. Infants who received PDA treatment based solely on clinical diagnosis without echocardiographic confirmation or who underwent primary procedural closure were excluded. Prior to study initiation, each NICU self-selected 1 of 4 predefined interventions (standard-dose ibuprofen, adjustable-dose ibuprofen, indomethacin, and acetaminophen) as their primary pharmacotherapy based on a consensus decision of neonatologists practicing at their respective sites (eFigure 1 in Supplement 1).

### Outcomes

The primary outcome was failure of primary pharmacotherapy, defined as need for additional medical and/or surgical or interventional treatment following initial medication administration. The secondary outcomes included receipt of repeat pharmacotherapy course; surgical or interventional PDA closure; moderate to severe bronchopulmonary dysplasia (BPD), defined as oxygen or respiratory support requirement at 36 weeks postmenstrual age or at discharge^8^; stage 2 or higher necrotizing enterocolitis (NEC)^9^; severe (grade III-IV) IVH^10^; definite sepsis (positive bacterial culture in blood or cerebrospinal fluid)^11^; stage 1 or higher acute kidney injury^12^; posttreatment peak serum bilirubin, aspartate aminotransferase, and alanine aminotransferase within 1 week of treatment initiation; and all-cause in-hospital mortality.

### Study Implementation and Data Collection

Trained data abstractors reviewed echocardiography reports for all potentially eligible infants. If a PDA was documented, those records were reviewed by the respective site investigators for eligibility. Eligible infants were identified through documentation of a moderate- to large-sized (≥1.5 mm) predominantly left-to-right shunting PDA on echocardiography. Infants were excluded if they had a clinical condition that would preclude medical treatment of PDA, such as being moribund; a decision to withdraw life-sustaining therapies, a small or tiny PDA, or clinical or echocardiographic features suggestive of acute pulmonary hypertension. All clinical decisions, including obtaining echocardiography, patient selection for treatment, and subsequent management plans, were made per the discretion of the medical team.

### Sample Size

Sample size estimation was based on the primary outcome. The best estimates of pharmacotherapy failure reported in RCTs range from 26% to 29%.^4,6^ However, data from Canadian observational studies suggest that primary pharmacotherapy failure rate with standard-dose ibuprofen, the most common regimen, is about 40%.^5,13^ To identify a reduction in pharmacotherapy failure rate from 40% to 26% for adjustable-dose ibuprofen compared with the other regimens, with 80% power and 2-sided significance level of *P* = .05, we required at least 263 infants in the adjustable-ibuprofen group and 198 infants each in the other treatment groups. Overall, we aimed to include approximately 850 infants who received PDA treatment as well as approximately 500 infants who did not receive PDA treatment, for a total sample of 1350 infants.

### Statistical Analysis

We planned for a primary intention-to-treat (ITT) analysis (site-level analysis) and a secondary drug-dose effectiveness analysis (patient-level analysis) a priori. In the ITT analysis, infants were classified into 4 treatment groups (standard-dose ibuprofen, adjustable-dose ibuprofen, indomethacin, and acetaminophen) in accordance with the treatment assignment by NICUs and not the actual drug dose received by patients. In the drug-dose effectiveness analysis, infants were grouped based on the actual drug regimen they received during the first course of treatment.

To determine the relative effectiveness of the 4 treatment groups, we applied multiple logistic regression models that were adjusted for gestational age (GA), sex, small for GA (SGA) status, outborn status, antenatal corticosteroids use, multiple births, Respiratory Severity Score (mean airway pressure by fraction of inspired oxygen) at treatment initiation, pretreatment PDA size (millimeters), and age at treatment (days). To account for clustering within units, we used the generalized estimating equations (GEE) with standard robust (empirical) variance estimate and with site as a clustering variable.

To test the robustness of the findings, we conducted an inverse probability weighting (IPW) analysis.^14^ The generalized propensity score (defined as the conditional probability of receiving a particular treatment) was first estimated using a multinomial logistic regression model for receipt of treatments (4-level dependent variable) on covariates (independent variables). Next, we used multiple weighted logistic regression models for each outcome (where the weight was defined as the inverse of the generalized propensity score) to examine the difference in the relative effectiveness between treatment groups.

A per-protocol sensitivity analysis was conducted post hoc after removing infants with protocol deviation from the ITT cohort. Subgroup analyses were conducted based on timing of treatment (<7 vs ≥7 days) and GA cutoff (<26 vs ≥26 weeks’ gestation), and sensitivity analysis for GEE models adjusted for variance estimates accounting for a small number of clusters (n = 19 sites) was performed.

A 2-sided *P* < .05 was considered to be statistically significant. Analyses were conducted from May 9, 2024, to February 23, 2026, using SAS, version 9.4 (SAS Institute Inc) and R, version 3.4.446 (R Project for Statistical Computing).

## Results

A total of 1356 infants (mean [SD] GA, 25.4 [1.6] weeks; mean [SD] birth weight, 828 [228] g; 746 males [55.0%] and 610 females [45.0%]) were included between January 2020 and July 2023. Of these patients, 1097 (80.9%) received PDA pharmacotherapy, whereas 259 (19.1%) did not receive pharmacotherapy (or were conservatively treated) (Figure). Among the participating NICUs, 8 intended to use standard-dose ibuprofen, 8 selected adjustable-dose ibuprofen, 3 selected acetaminophen, and 1 selected indomethacin. One site intending to use standard-dose ibuprofen was excluded due to having fewer than 5 eligible participants.

**Figure.**
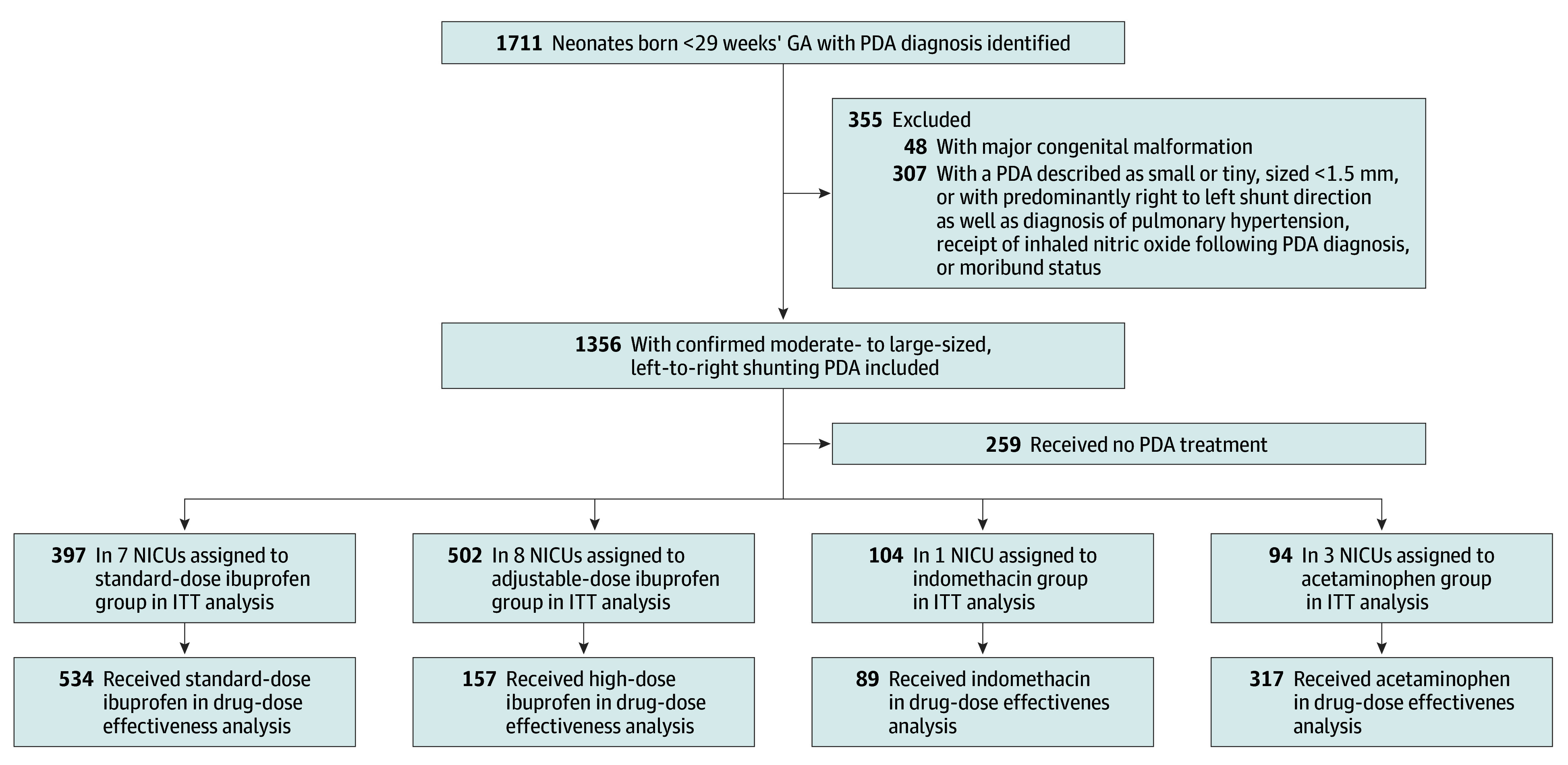
Study Flowchart GA indicates gestational age; NICU, neonatal intensive care unit; and PDA, patent ductus arteriosus.

### Determination of Treatment Cohorts

#### ITT Cohort

Based on the intended choice of primary pharmacotherapy by each NICU, 397 eligible infants (7 sites) were included in the standard-dose ibuprofen arm, 502 infants (8 sites) in the adjustable-dose ibuprofen arm, 104 infants (1 site) in the indomethacin arm, and 94 infants (3 sites) in the acetaminophen arm. These infants constituted the ITT cohort (Figure).

#### Drug-Dose Effectiveness Cohort

The actual primary pharmacotherapy administered to each infant was the basis for the drug-dose effectiveness cohort: 691 infants received ibuprofen, 89 received indomethacin, and 317 received acetaminophen (Figure; Table 1). Of the 691 infants who received ibuprofen, 658 (95.2%) had their dose documented. The mean (SD) initial ibuprofen dose was 12.4 (6.5) mg/kg. Based on the distribution of ibuprofen dose, an initial dose higher than 12.5 mg/kg was defined as the cutoff for high-dose ibuprofen. Of the remaining 33 infants without a recorded dose, the treatment arms were imputed based on the initial assignment. Based on these assignments, the drug-dose effectiveness cohort constituted 534 infants in the standard-dose ibuprofen arm and 157 infants in the adjustable-dose ibuprofen arm (all of whom received high-dose ibuprofen).

**Table 1.** Baseline Demographic Characteristics of the Drug-Dose Effectiveness Cohort

Characteristic	Infants, No. (%)				
	Ibuprofen		Indomethacin (n = 89)	Acetaminophen (n = 317)	PDA conservatively managed (n = 259)
	Standard dose (n = 534)	Adjustable dose (n = 157)			
GA at birth, median (IQR), wk	25 (24-26)	25 (24-27)	25 (24-26)	25 (24-26)	26 (25-27)
GA <26 wk at birth	324 (60.7)	86 (54.8)	46 (51.7)	195 (61.5)	90 (34.8)
Birth weight, mean (SD), g	803 (215)	824 (237.4)	822 (197)	782 (213)	938 (245)
Sex					
Male	303 (56.7)	88 (56.1)	41 (46.1)	183 (57.7)	131 (50.6)
Female	231 (43.3)	69 (43.9)	48 (53.9)	134 (42.3)	128 (49.4)
SGA status^a^	59 (11.1)	14 (8.9)	7 (7.9)	48 (15.1)	17 (6.6)
Outborn status	64 (12)	22 (14)	20 (22.5)	59 (18.6)	46 (17.8)
Apgar score at 5 min, median (IQR)	6 (5-8)	7 (5-8)	8 (5-8)	6 (4-8)	7 (5-9)
Received >1 dose of surfactant	264 (49.4)	56 (35.7)	31 (34.8)	133 (42.0)	80 (30.9)
SNAP-II score >20	179 (33.8)	45 (28.7)	34 (38.2)	105 (33.1)	62 (23.9)
Age at first treatment, median (IQR), d	5 (3-8)	12 (9-18)	5 (2-8)	8 (5-17)	NA
PDA size, median (IQR), mm^b^	2.0 (1.7-2.5)	2.1 (1.7-2.4)	2.0 (2.0-2.5)	2.2 (1.9-2.6)	1.7 (1.4-2.1)
RSS, median (IQR)^c^	2.4 (1.9-4.2)	2.5 (2.0-3.5)	2.2 (1.7-2.9)	2.8 (2.1-4.2)	1.92 (1.5-3.0)

Abbreviations: GA, gestational age; NA, not applicable; PDA, patent ductus arteriosus; RSS, respiratory severity score; SGA, small for gestational age; SNAP-II, Score for Neonatal Acute Physiology, revised version.

^a^ Defined as infant weight below the 10th percentile of weight for the GA.

^b^ Estimated at the time of pretreatment echocardiography for treated infants or first echocardiography for untreated infants.

^c^ Assessed at the time of pretreatment echocardiography for treated infants or first echocardiography for untreated infants.

Overall, there was 28.1% protocol deviation (308 of 1097), 75.0% of which was attributable to acetaminophen use by sites that did not intend to use acetaminophen; the actual treatment choice by site is shown in eFigure 2 in Supplement 1. Given the substantial protocol deviation, we made a post hoc decision to conduct all primary analyses using the drug-dose effectiveness cohort. The baseline characteristics of the drug-dose effectiveness cohort are provided in Table 1. Among these infants, the median (IQR) age of pharmacotherapy initiation ranged from 5 (2-8) to 12 (9-18) days. eTables 3 and 6 in Supplement 1 provide baseline demographic characteristics of the ITT and per-protocol cohorts.

### Treatment Effectiveness

Overall, among the 1097 infants who received PDA treatment, 464 (42.3%) experienced failure of the primary pharmacotherapy, 456 (41.6%) received repeat pharmacotherapy course, and 45 (4.1%) underwent interventional PDA closure (Table 2). There were no differences between the 4 regimens in failure of primary pharmacotherapy or use of repeat treatment when adjusted for confounders and accounting for clustering within each site (Table 3; eTable 1 in Supplement 1). With regard to secondary outcomes, compared with the standard-dose ibuprofen arm, mortality odds were higher among infants who received acetaminophen (adjusted odds ratio [AOR], 1.37; 95% CI, 1.02-1.85) and lower among those who received indomethacin (AOR, 0.40; 95% CI, 0.16-0.99); sepsis odds were also lower among those who received indomethacin (AOR, 0.21; 95% CI, 0.07-0.63), while interventional PDA closure odds were lower among those who received adjustable-dose ibuprofen (AOR, 0.30; 95% CI, 0.16-0.59) (Table 3). Sensitivity analyses using IPW models demonstrated consistent results with the primary outcome (Table 3). No differences in adverse effects (posttreatment serum creatinine, bilirubin, and liver function) were noted with any of the pharmacotherapeutic options (eTable 2 in Supplement 1).

**Table 2.** Overall Clinical Outcomes in Infants Receiving Patent Ductus Arteriosus Treatment

Outcome	Infants, No./Total No. (%)
Primary	
Failure of primary pharmacotherapy	464/1097 (42.3)
Secondary	
Repeat pharmacotherapy course	456/1097 (41.6)
Interventional PDA closure	45/1097 (4.1)
Predischarge mortality	159/1097 (14.5)
Moderate to severe BPD	606/919 (65.9)
NEC stage ≥2	127/1097 (11.6)
Definite sepsis following initiation of treatment	250/1097 (22.8)

Abbreviations: BPD, bronchopulmonary dysplasia; NEC, necrotizing enterocolitis; PDA, patent ductus arteriosus.

**Table 3.** Modeled Estimates for Study Outcomes of the Drug-Dose Effectiveness Cohort

Outcome	AOR (95% CI)					
	Model 1^a^			Model 2^b^		
	Adjustable-dose ibuprofen	Indomethacin	Acetaminophen	Adjustable-dose ibuprofen	Indomethacin	Acetaminophen
Failure of primary pharmacotherapy	1.24 (0.78-1.96)	0.94 (0.42-2.09)	0.94 (0.59-1.49)	1.13 (0.57-2.23)	1.04 (0.32-3.39)	1.23 (0.72-2.08)
Repeat pharmacotherapy course	1.32 (0.79-2.21)	1.13 (0.53-2.39)	0.84 (0.51-1.41)	1.19 (0.60-2.36)	1.07 (0.33-3.44)	1.11 (0.66-1.85)
Interventional PDA closure	0.30 (0.16-0.59)^c^	0.39 (0.11-1.38)	0.76 (0.46-1.25)	0.80 (0.35-1.83)	0.39 (0.09-1.79)	1.18 (0.66-2.13)
Predischarge mortality	1.43 (0.60-3.42)	0.40 (0.16-0.99)^c^	1.37 (1.02-1.85)^d^	1.53 (0.78-3.00)	0.21 (0.04-1.30)	1.42 (0.85-2.40)
Moderate to severe BPD	0.86 (0.41-1.80)	1.27 (0.76-2.11)	0.84 (0.51-1.38)	0.37 (0.21-0.64)^c^	3.56 (1.82-6.99)^d^	0.63 (0.39-1.01)
NEC stage ≥2	1.07 (0.63-1.82)	0.91 (0.49-1.67)	1.49 (0.88-2.51)	1.28 (0.58-2.85)	2.27 (0.89-5.83)	1.49 (0.88-2.50)
Definite sepsis	1.15 (0.73-1.82)	0.21 (0.07-0.63)^c^	1.00 (0.66-1.52)	1.25 (0.64-2.42)	0.04 (0.01-0.25)^c^	0.84 (0.50-1.43)

Abbreviations: AOR, adjusted odds ratio; BPD, bronchopulmonary dysplasia; NEC, necrotizing enterocolitis; PDA, patent ductus arteriosus.

^a^ Model 1: AORs were computed using multiple logistic regression models, with a generalized estimating equation approach accounting for clustering within each site, and adjusted for gestational age (GA), sex, small for gestational age (SGA) status, outborn status, antenatal corticosteroids use, multiple births, respiratory severity score (RSS), and pretreatment PDA size. Standard-dose ibuprofen was the reference group.

^b^ Model 2: AORs were computed using inverse probability weighting analysis, which estimated the probability of the primary exposure based on GA, sex, SGA status, outborn status, antenatal corticosteroids use, multiple births, RSS, and pretreatment PDA size. Stabilization using marginal probability on weights was applied. Standard-dose ibuprofen was the reference group.

^c^ Demonstrates a statistically significant benefit at *P* < .05. *P* value was not adjusted for multiple comparisons.

^d^ Demonstrates a statistically significant harm at *P* < .05. *P* value was not adjusted for multiple comparisons.

When compared with those who received no pharmacologic treatment, infants who received PDA pharmacotherapy had higher odds of moderate to severe BPD (AOR, 1.91; 95% CI, 1.12-3.25) and NEC (AOR, 2.15; 95% CI, 1.55-2.99) (Table 4). The IPW model, which also demonstrated similar results, also showed lower mortality odds in the PDA-treated group (AOR, 0.35; 95% CI, 0.21-0.58).

**Table 4.** Clinical Outcomes of Infants Receiving Patent Ductus Arteriosus Treatment vs Conservative Management

Clinical outcome	Infants, No. (%)		AOR (95% CI)	
	PDA treated with pharmacotherapeutics (n = 1097)	PDA conservatively managed (n = 259)	Model 1^a^	Model 2^b^
Predischarge mortality	159 (14.5)	40 (15.4)	0.59 (0.34-1.05)	0.35 (0.21-0.58)^c^
Moderate to severe BPD	606 (55.2)	84 (32.4)	1.91 (1.12-3.25)^d^	2.11 (1.29-3.45)^d^
NEC stage ≥2	127 (11.6)	16 (6.2)	2.15 (1.55-2.99)^d^	2.05 (1.33-3.17)^d^
Definite sepsis	250 (22.8)	52 (21.2)	1.57 (0.98-2.51)	1.37 (0.61-3.06)

Abbreviations: AOR, adjusted odds ratio; BPD, bronchopulmonary dysplasia; NEC, necrotizing enterocolitis; PDA, patent ductus arteriosus.

^a^ Model 1: AORs were computed using multiple logistic regression models, with generalized estimating equation approach accounting for clustering within each site, and adjusted for gestational age (GA), sex, small for gestational age (SGA) status, outborn status, antenatal corticosteroids use, multiple births, respiratory severity score (RSS), and pretreatment PDA size.

^b^ Model 2: AORs were computed using inverse probability weighting analysis. Propensity score estimated the predictive probability of the primary exposure based on GA, sex, SGA status, outborn status, antenatal corticosteroids use, multiple births, RSS, and pretreatment PDA size. Stabilization using marginal probability on weights was applied.

^c^ Demonstrates a statistically significant benefit at *P* < .05. *P* value was not adjusted for multiple comparisons.

^d^ Demonstrates a statistically significant harm at *P* < .05. *P* value was not adjusted for multiple comparisons.

Post hoc per-protocol sensitivity and subgroup analyses showed no difference in results for the primary outcome (eTables 3-11 in Supplement 1). Per-protocol sensitivity analyses were largely consistent with the primary analysis, demonstrating higher mortality odds with acetaminophen (AOR, 1.47; 95% CI, 1.12- 1.92) and lower mortality (AOR, 0.16; 95% CI, 0.14-0.18), lower sepsis (AOR, 0.07; 95% CI, 0.04-0.10), and higher BPD odds (AOR, 2.06; 95% CI, 1.24-3.44) with indomethacin (eTable 8 in Supplement 1). Notable findings from subgroup analyses include lower mortality odds with indomethacin within 7 days of birth (AOR, 0.16; 95% CI, 0.03-0.96) and higher BPD odds with indomethacin both within (AOR, 2.69; 95% CI, 1.19-6.11) and after 7 days (AOR, 7.67; 95% CI, 1.75-33.74) (eTable 9 in Supplement 1). In addition, in the subgroup of infants born before 26 weeks’ gestation, indomethacin was associated with lower mortality (AOR, 0.10; 95% CI, 0.01-0.82) and higher BPD odds (AOR, 11.20; 95% CI, 2.93-42.95), while adjustable-dose ibuprofen was associated with lower BPD odds in both GA subgroups (<26 weeks GA: AOR, 0.33 [95% CI, 0.14-0.75]; ≥26 weeks GA: AOR, 0.38 [95% CI, 0.18-0.82]) (eTable 10 in Supplement 1).

## Discussion

In this multicenter, prospective, comparative effectiveness research study of extremely preterm infants with a moderate- to large-sized PDA, failure of primary pharmacotherapy was high regardless of medication choice. Our results offer insights into response to PDA medical therapy that complement existing evidence from RCTs. In this study, the median age of therapy initiation ranged from 5 to 12 days, and the overall use of repeat treatment following failure of primary pharmacotherapy was high (41.6%). In contrast, a recent Cochrane review of early PDA treatment vs expectant management identified a repeat medical therapy rate following failure of primary pharmacotherapy of only 19.5% when treatment was initiated within the first 3 days of birth.^11^ Furthermore, in 2 other RCTs where PDA treatment was initiated between 6 and 14 days of age, the primary pharmacotherapy failure rates were noted to be 57% and 80%.^15,16^ These findings suggest that earlier initiation of therapy within the first 3 days of life may provide a higher PDA closure success rate, while choice of therapy may have only a small role in therapy success if initiated beyond the first few days of life, with high failure rates regardless of treatment choices. This information may have important implications for a clinician’s decision on timing of PDA management.

With regard to secondary outcomes, our result suggesting increased mortality odds with acetaminophen use aligns with a recent report from the National Institute of Child Health and Human Development Neonatal Research Network (adjusted risk ratio, 1.42; 95% CI, 1.02-1.93).^17^ This previous study had missing information on timing of PDA treatment, thereby leading to uncertainty on whether some covariates were true confounders or occurred after treatment. In contrast, our prospective study had detailed information on the timing of PDA diagnosis and treatment; therefore, we are confident that the covariates included in our regression models were true confounders. However, we acknowledge that unaccounted confounding by indication and contraindication cannot be ruled out as we did not record precise timing and occurrence of other clinical parameters that might affect the choice of acetaminophen, such as pretreatment platelet counts, feed tolerance, gastrointestinal perforation, and severe IVH diagnosis based on early cranial ultrasonography.

In our study, indomethacin was associated with a large reduction in odds of mortality and sepsis, 2 of the most patient-important outcomes. However, the results should be interpreted with caution as such implausible effect sizes might represent a statistical fallacy driven by outcomes of a single NICU with a relatively small number of participants (n = 89) who used indomethacin as their primary pharmacotherapy.

We found that infants who received PDA treatment had higher odds of moderate to severe BPD and NEC compared with infants who were treated conservatively. Our results align with findings from recent large RCTs on early PDA therapy. Both the Beneductus and Baby-OSCAR trials showed higher rates of BPD with early ibuprofen use in extremely preterm infants.^18,19^ On the contrary, while meta-analysis of recent RCTs demonstrated an increase in mortality with early ibuprofen use (risk ratio, 1.36; 95% CI, 1.03-1.79),^11^ our study found a potential survival advantage in infants who received PDA treatment despite being smaller and sicker than infants who did not receive pharmacotherapy. This divergence between trial data and clinical data has previously been noted in several large population-based cohorts. For example, in a French cohort of extremely preterm infants, screening echocardiography and PDA treatment before day 3 of life were associated with lower mortality (OR, 0.73; 95% CI, 0.54-0.98)^20^; the recent follow-up cohort demonstrated a persistent survival benefit at 5.5 years (OR, 1.40; 95% CI, 1.05-1.88), with possible improvement in survival without moderate to severe neurodevelopmental disabilities (OR, 1.29; 95% CI, 1.00-1.68).^21^ Similarly, a retrospective repeated-measures study of very low birth weight infants from the US (n = 32 094 between 2008 and 2015) showed a 0.21–percentage point increase in mortality for each interepoch decrease of 1.00 percentage point in medical or surgical PDA treatment.^22^ While the recent RCTs that demonstrated potential harm with COX inhibitors focused on early presymptomatic PDA therapy,^18,19^ in the clinical studies that suggested a mortality benefit, symptomatic infants beyond the initial transition period were primarily exposed to PDA therapy,^22,23^ in alignment with our findings. Consequently, it is not surprising that a large cohort study (n = 39 096) across 139 NICUs found that both low and high PDA treatment rates were associated with death or severe neurologic injury.^23^ Therefore, existing observational data, in conjunction with our findings, suggest the need for a more judicious approach to selecting patients for PDA pharmacotherapy.

### Limitations

This study has limitations. First, it was not an RCT; therefore, self-selection of treatment strategies by NICUs, potential bias arising from unblinded case adjudication, and clinician discretion with regard to treatment thresholds may have resulted in systematic differences in clinical characteristics of enrolled participants. Furthermore, no standardized CNN-wide protocol was used for echocardiographic measurements; hence, diagnostic precision could not be ensured across sites. Both our primary (multivariable logistic regression with GEE) and secondary (IPW) analytical models accounted for such potential differences in baseline confounders, and the GEE model specifically accounted for site-level factors. However, we cannot confidently rule out unmeasured confounders, including site-specific diagnostic and clinical practices. Second, there was a large deviation from self-selected protocols related to acetaminophen use by sites that did not intend to use acetaminophen. As a result, a post hoc decision was made to conduct the primary analyses on the patient-level drug-dose effectiveness cohort rather than the site-level ITT cohort. Therefore, we did not use intracluster correlation coefficient adjustment to account for the lack of independence among infants within the same site. We acknowledge that this decision may have introduced a type I error due to our choice of analytical model. Reassuringly, on sensitivity analysis of the GEE model accounting for clustering within each site and using a small sample–corrected empirical variance estimator (Mancl and DeRouen correction), our results were largely in alignment with results of the primary analysis. Furthermore, despite accounting for potential factors associated with acetaminophen as the primary pharmacotherapy choice (such as low GA and SGA status), unaccounted confounding by indication or contraindication cannot be ruled out. Hence, the association of acetaminophen with increased mortality odds should be interpreted with caution.

Third, we found notable differences in clinical outcomes (such as lower mortality and sepsis and higher BPD with indomethacin and lower interventional PDA closure with high-dose ibuprofen) without any plausible biologic rationale, given that primary treatment failure rates did not differ across groups. Moreover, there were inconsistencies in the effect estimates between analytic models. Hence, observed differences in clinical outcomes with different treatment regimens should be carefully interpreted as they might merely represent differences in statistical methodologies or center-level practices rather than true biologic variations. Fourth, there is a likelihood that the lower mortality odds associated with PDA treatment are attributable to survival bias. However, based on a thorough data evaluation of each included infant, we are confident that we had a homogenous cohort with a confirmed diagnosis of a moderate- to large-sized, left-to-right shunting PDA. All possible PDA cases for whom treatment was not possible nor indicated were excluded; therefore, the risk of survival bias was low. Finally, given that this study was conducted exclusively in Canadian NICUs, the results may not be generalizable to centers outside Canada with different health care delivery models.

## Conclusions

This large, multicenter comparative effectiveness research study showed that the primary PDA pharmacotherapy failure rate was high and similar in extremely preterm infants regardless of medication choices. Use of acetaminophen was associated with increased mortality odds, but this result should be interpreted with caution due to potential confounding by indication or contraindication. Although no pharmacotherapy choice stood out as more effective, overall infants who were exposed to pharmacotherapy had higher odds of moderate to severe BPD and NEC but lower mortality odds compared with infants with conservatively managed PDA. Future studies should investigate approaches for identifying extremely preterm infants at high risk for PDA-attributable morbidity and infants most likely to respond to medical therapy through a thorough examination of ancillary factors, such as biomarker profiles, echocardiographic PDA phenotypes, drug pharmacokinetics, and genetic factors, to maximize the benefits of and minimize unnecessary exposure to COX inhibitors.

## Notes

Supplement 1. **eFigure 1.** Study Schema **eFigure 2.** PDA Treatment Choice by Each Participating Site **eTable 1.** Crude Rates of Study Outcomes in Each Treatment Arm in the Drug-Dose Effectiveness Cohort **eTable 2.** Biochemical Measures of Adverse Effects **eTable 3.** Baseline Demographic Variables (Intention to Treat Cohort) **eTable 4.** Crude Rates of Study Outcomes in Each Treatment Arm in the Intention-to-Treat Cohort **eTable 5.** Modeled Estimates for Study Outcomes (Intention to Treat Cohort) **eTable 6.** Baseline Demographic Variables (Per-Protocol Cohort) **eTable 7.** Crude Rates of Study Outcomes in Each Treatment Arm in the Per-Protocol Cohort **eTable 8.** Modeled Estimates for Study Outcomes (Per-Protocol Cohort) **eTable 9.** Subgroup Analysis of the Drug Dose Effectiveness Cohort Based on Timing of Treatment **eTable 10.** Subgroup Analysis of the Drug Dose Effectiveness Cohort Based on Gestational Age Cut-off **eTable 11.** Sensitivity Analysis of the GEE Models Adjusting for Variance Estimates (Accounting for the Small Number of Clusters)

Supplement 2.
Nonauthor Collaborators

Supplement 3.
Data Sharing Statement
