# Supplementary material for: Pharmacologic Therapies for Patent Ductus Arteriosus in Extremely Preterm Infants
Source: JAMA Netw Open. 2026 Jun 9;9(6):e2617477. doi: 10.1001/jamanetworkopen.2026.17477 (PMC13250707; doi:10.1001/jamanetworkopen.2026.17477)
Supplement: Supplement 1. — eFigure 1. Study Schema eFigure 2. PDA Treatment Choice by Each Participating Site eTable 1. Crude Rates of Study Outcomes in Each Treatment Arm in the Drug-Dose Effectiveness Cohort eTable 2. Biochemical Measures of Adverse Effects eTable 3. Baseline Demographic Variables (Intention to Treat Cohort) eTable 4. Crude Rates of Study Outcomes in Each Treatment Arm in the Intention-to-Treat Cohort eTable 5. Modeled Estimates for Study Outcomes (Intention to Treat Cohort) eTable 6. Baseline Demographic Variables (Per-Protocol Cohort) eTable 7. Crude Rates of Study Outcomes in Each Treatment Arm in the Per-Protocol Cohort eTable 8. Modeled Estimates for Study Outcomes (Per-Protocol Cohort) eTable 9. Subgroup Analysis of the Drug Dose Effectiveness Cohort Based on Timing of Treatment eTable 10. Subgroup Analysis of the Drug Dose Effectiveness Cohort Based on Gestational Age Cut-off eTable 11. Sensitivity Analysis of the GEE Models Adjusting for Variance Estimates (Accounting for the Small Number of Clusters) [file jamanetwopen-e2617477-s001.pdf]

# Supplemental Online Content

Mitra S, Jain A, Ting JY, et al; for the Canadian Neonatal Network Investigators. Pharmacologic therapies for patent ductus arteriosus in extremely preterm infants. *JAMA Netw Open*. 2026;9(6):e2617477. doi:10.1001/jamanetworkopen.2026.17477

**eFigure 1.** Study Schema

**eFigure 2.** PDA Treatment Choice by Each Participating Site

**eTable 1.** Crude Rates of Study Outcomes in Each Treatment Arm in the Drug-Dose Effectiveness Cohort

**eTable 2.** Biochemical Measures of Adverse Effects

**eTable 3.** Baseline Demographic Variables (Intention to Treat Cohort)

**eTable 4.** Crude Rates of Study Outcomes in Each Treatment Arm in the Intention-to-Treat Cohort

**eTable 5.** Modeled Estimates for Study Outcomes (Intention to Treat Cohort)

**eTable 6.** Baseline Demographic Variables (Per-Protocol Cohort)

**eTable 7.** Crude Rates of Study Outcomes in Each Treatment Arm in the Per-Protocol Cohort

**eTable 8.** Modeled Estimates for Study Outcomes (Per-Protocol Cohort)

**eTable 9.** Subgroup Analysis of the Drug Dose Effectiveness Cohort Based on Timing of Treatment

**eTable 10.** Subgroup Analysis of the Drug Dose Effectiveness Cohort Based on Gestational Age Cut-off

**eTable 11.** Sensitivity Analysis of the GEE Models Adjusting for Variance Estimates (Accounting for the Small Number of Clusters)

This supplemental material has been provided by the authors to give readers additional information about their work.

© 2026 Mitra S et al. *JAMA Network Open.*

**eFigure 1. Study Schema**

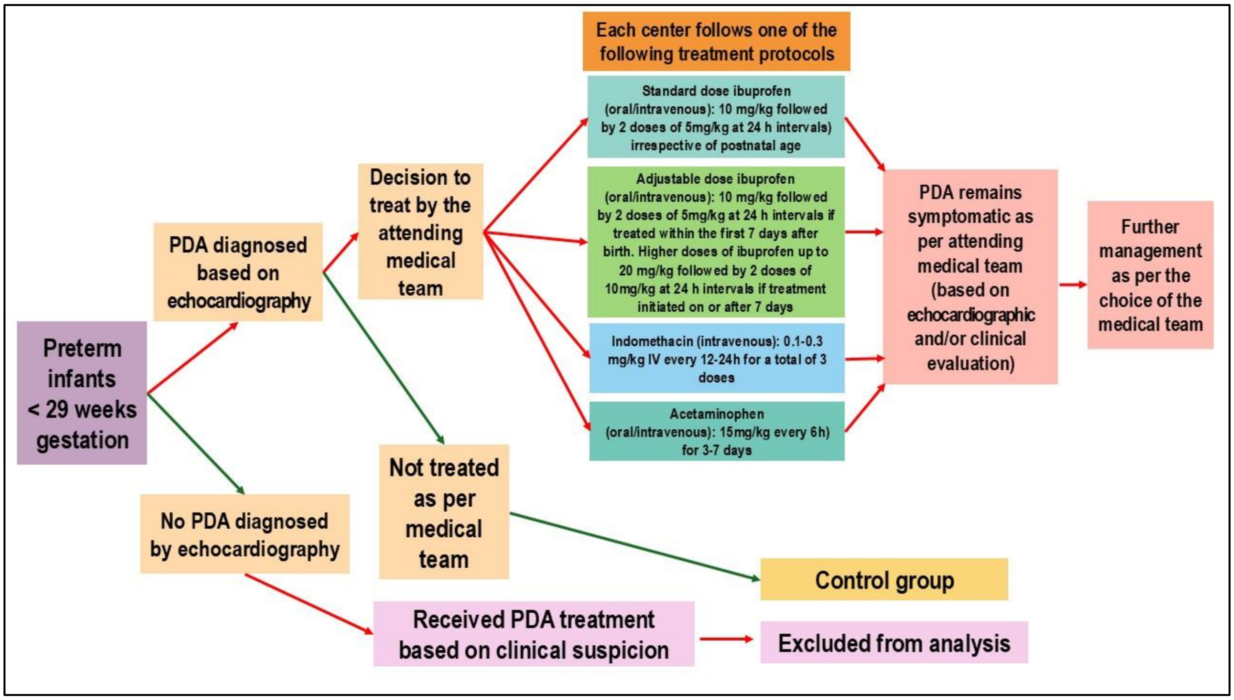


PDA, patent ductus arteriosus

© 2026 Mitra S et al. *JAMA Network Open.*

## eFigure 2. PDA treatment choice by each participating site

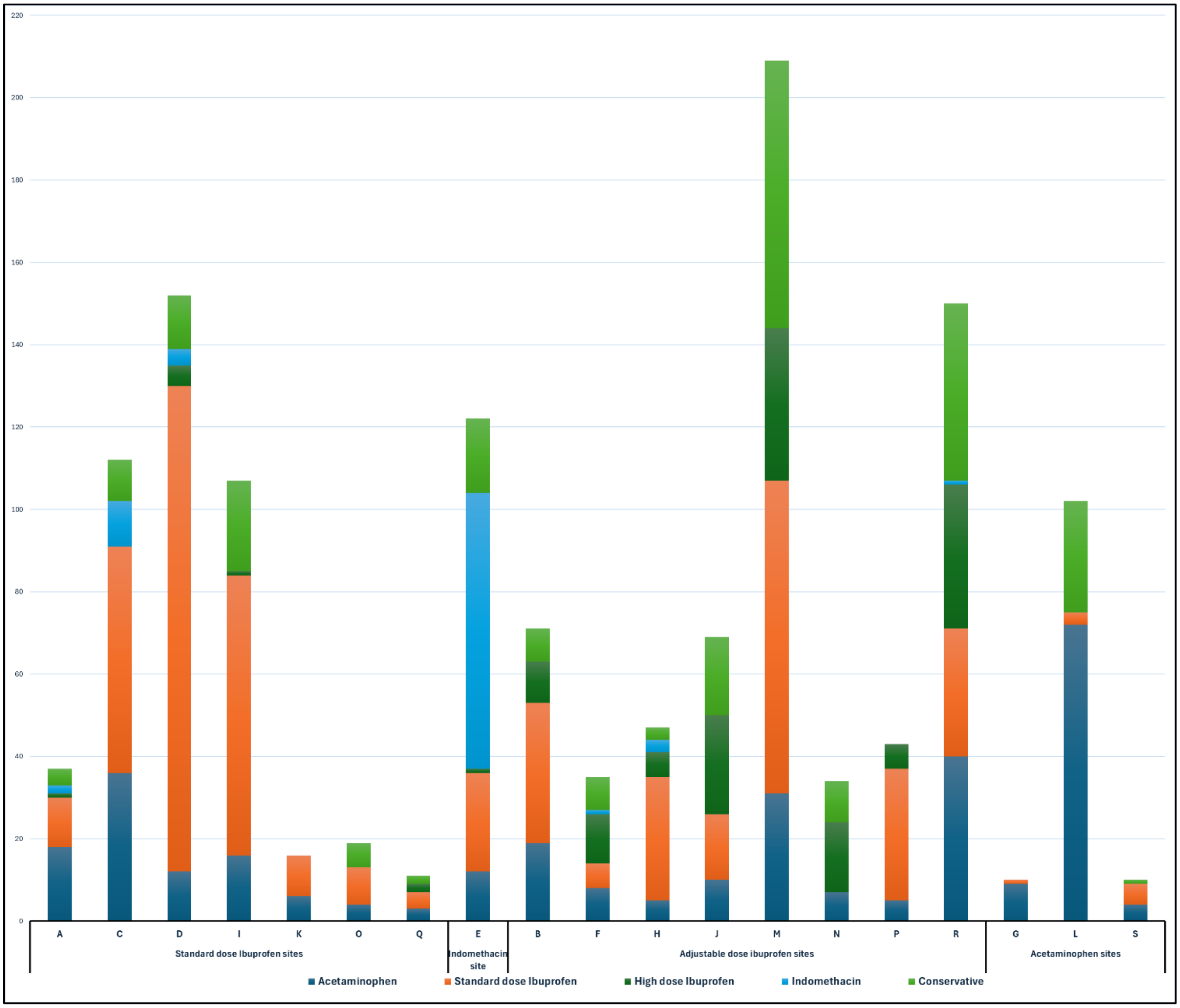


The figure demonstrates actual initial PDA pharmacotherapy received at each site. The sites have been grouped together as per their initial choice of pharmacotherapy

© 2026 Mitra S et al. *JAMA Network Open.*

**eTable 1. Crude rates of study outcomes in each treatment arm in the drug-dose effectiveness cohort**

| Outcomes | Standard dose ibuprofen (N=534) | Adjustable dose ibuprofen (N=157) | Indomethacin (N=89) | Acetaminophen (N=317) |
|---|---|---|---|---|
| **Primary outcome, n (%)** | | | | |
| Failure of primary pharmacotherapy | 225 (42.1) | 77 (49.0) | 35 (39.3) | 133 (42.0) |
| **Secondary outcomes, n (%)** | | | | |
| Repeat medical therapy | 221 (41.4) | 77 (49.0) | 35 (39.3) | 123 (38.8) |
| Interventional PDA closure | 52 (9.7) | 11 (7.0) | 8 (9.0) | 36 (11.4) |
| Pre-discharge mortality | 82 (15.4) | 19 (12.1) | 6 (6.7) | 52 (16.4) |
| Moderate-Severe BPD | 302 (56.4) | 73 (45.9) | 69 (77.5) | 162 (48.3) |
| NEC (stage 2 or greater) | 61 (11.4) | 14 (8.9) | 7 (7.9) | 45 (14.2) |
| Definite sepsis following initiation of treatment | 142 (26.6) | 31 (19.8) | 5 (5.7) | 72 (22.7) |

© 2026 Mitra S et al. *JAMA Network Open.*

**eTable 2. Biochemical measures of adverse effects**

| Outcomes | Standard dose ibuprofen (N=534) | Adjustable dose ibuprofen (N=157) | Indomethacin (N=67) | Acetaminophen (N=94) |
|---|---|---|---|---|
| **Max creatinine post treatment (u/L)**; Median (IQR) | 71 (59, 86) | 57 (45, 74) | 72 (62, 92) | 63 (45, 83) |
| **Post-treatment serum bilirubin** (maximum serum bilirubin (uMol/L) within 1 week of initiation of each course of pharmacotherapy); Median (IQR) | 112.5 (88, 141) | 85 (48, 123) | 119 (99, 139) | 103 (50, 134) |
| **Maximum serum AST (u/L)** during treatment or within 1 week of treatment completion; Median (IQR) | Not measured | Not measured | Not measured | 32 (24, 50) |
| **Maximum serum ALT (u/L)** during treatment or within 1 week of treatment completion; Median (IQR) | Not measured | Not measured | Not measured | 9 (7, 14.5) |

© 2026 Mitra S et al. *JAMA Network Open.*

**eTable 3. Baseline demographic variables (intention to treat cohort)**

| Variables | Standard dose ibuprofen (n=397) | Adjustable dose ibuprofen (n=502) | Indomethacin (n=104) | Acetaminophen (n=94) | PDA not treated (n=259) |
|---|---|---|---|---|---|
| Gestational age at birth (weeks); Median, IQR | 25 (24, 27) | 25 (24, 26) | 25 (24, 26) | 25 (24, 26) | 26 (25, 27) |
| Gestational age <26 weeks at birth; N (%) | 220 (55.4) | 313 (62.4) | 57 (54.8) | 61 (64.9) | 90 (34.8) |
| Birth weight (grams); Mean (SD) | 833 (216) | 777 (216) | 808 (204) | 800 (222) | 938 (245) |
| Male sex; N (%) | 235 (59.2) | 279 (55.6) | 46 (44.2) | 55 (58.5) | 131 (50.6) |
| Small for gestational age (<10th percentile); N (%) | 39 (9.8) | 70 (13.9) | 9 (8.7) | 10 (10.6) | 17 (6.6) |
| Outborn; N (%) | 54 (13.6) | 72 (14.3) | 17 (16.4) | 22 (23.4) | 46 (17.8) |
| Apgar score at 5 min; Median (IQR) | 6 (5, 7) | 7 (5, 8) | 7 (6, 8) | 6 (3, 8) | 7 (5, 9) |
| Number of patients who received >1 dose of surfactant, n (%) | 191 (48.1) | 220 (43.8) | 38 (36.5) | 35 (37.2) | 80 (30.9) |
| SNAPII score >20, n (%) | 154 (39.3) | 157 (31.3) | 35 (33.7) | 17 (18.1) | 62 (23.9) |
| Age at first treatment (days), median, IQR | 5 (3, 9) | 7 (4, 12) | 8 (5, 13) | 11 (7, 21) | NA |
| PDA size* (mm); Median, IQR | 2.1 (1.8, 2.5) | 2.1 (1.7, 2.4) | 2.0 (2.0, 2.5) | 2.2 (2.0, 2.7) | 1.7 (1.4, 2.1) |
| Respiratory severity score**; Median, IQR | 2.5 (1.9, 3.6) | 2.5 (2.0, 3.4) | 2.1 (1.5, 2.7) | 3.5 (2.5, 4.4) | 1.92 (1.5 3.0) |

NA: Not applicable

*Estimated PDA size on (a) pre-treatment echocardiography for treated infants; or (b) first echocardiography for untreated infants

** Respiratory severity score assessed at time of (a) pre-treatment echocardiography for treated infants; or (b) first echocardiography for untreated infants

© 2026 Mitra S et al. *JAMA Network Open.*

**eTable 4. Crude rates of study outcomes in each treatment arm in the intention-to-treat cohort**

| Outcomes | Standard dose ibuprofen (N=397) | Adjustable dose ibuprofen (N=502) | Indomethacin (N=104) | Acetaminophen (N=94) |
|---|---|---|---|---|
| **Primary outcome, n (%)** | | | | |
| Failure of primary pharmacotherapy | 165 (41.6) | 221 (44.0) | 38 (36.5) | 46 (48.9) |
| **Secondary outcomes, n (%)** | | | | |
| Repeat medical therapy | 162 (40.8) | 216 (43.0) | 34 (32.7) | 44 (46.8) |
| Interventional PDA closure | 31 (7.8) | 60 (12.0) | 12 (11.5) | 4 (4.3) |
| Pre-discharge mortality | 56 (14.1) | 88 (17.5) | 3 (2.9) | 12 (12.8) |
| Moderate-Severe BPD | 250 (73.1) | 228 (58.2) | 85 (83.3) | 43 (51.8) |
| NEC (stage 2 or greater) | 42 (10.6) | 65 (13.0) | 9 (8.7) | 11 (11.7) |
| Definite sepsis following initiation of treatment | 100 (25.2) | 118 (23.5) | 8 (7.8) | 24 (25.5) |

© 2026 Mitra S et al. *JAMA Network Open.*

## eTable 5. Modeled estimates for study outcomes (intention to treat cohort)

| | Model 1: Logistic regression model with GEE[a] | | | Model 2: Inverse Probability Weighted model[b] | | |
|---|---|---|---|---|---|---|
| | **Adjustable dose ibuprofen** | **Indomethacin** | **Acetaminophen** | **Adjustable dose ibuprofen** | **Indomethacin** | **Acetaminophen** |
| **Outcomes** | *Adjusted OR (95% CI) with standard dose ibuprofen as reference[c]* | | | *Adjusted OR (95% CI) with standard dose ibuprofen as reference[c]* | | |
| **Failure of primary pharmacotherapy** | 1.06 (0.62, 1.80) | 0.76 (0.47, 1.20) | 1.36 (0.96, 1.93) | 1.1 (0.81, 1.51) | 1.09 (0.54, 2.21) | 1.57 (0.91, 2.72) |
| **Repeat medical therapy** | 1.06 (0.59, 1.90) | 0.62 (0.38, 1.03) | 1.41 (0.87, 2.29) | 1.14 (0.83, 1.57) | 0.86 (0.43, 1.71) | 1.71 (0.99, 2.95) |
| **Interventional PDA closure** | 1.86 (0.76, 4.56) | 2.46 (1.12, 5.37) | 0.4 (0.14, 1.14) | 1.9 (1.09, 3.33) | 2.2 (0.82, 5.86) | 0.39 (0.08, 1.81) |
| **Pre-discharge mortality** | 1.4 (0.85, 2.30) | 0.21 (0.15, 0.30) | 1.14 (0.65, 2.00) | 1.38 (0.90. 2.11) | 0.13 (0.02, 0.81) | 1.39 (0.59, 3.28) |
| **Moderate-Severe BPD** | 0.46 (0.21, 0.99) | 2.26 (1.40, 3.66) | 0.28 (0.13, 0.60) | 0.41 (0.28, 0.61) | 2.55 (1.27, 5.15) | 0.32 (0.16, 0.63) |
| **NEC (stage 2 or greater)** | 1.1 (0.56, 2.16) | 0.8 (0.41, 1.55) | 0.69 (0.36, 1.34) | 1.03 (0.64, 1.66) | 1.29 (0.47, 3.56) | 0.81 (0.27, 2.41) |
| **Definite sepsis** | 0.96 (0.49, 1.91) | 0.43 (0.25, 0.73) | 1.23 (0.60, 2.53) | 0.93 (0.65, 1.33) | 0.3 (0.10, 0.91) | 1.38 (0.69, 2.75) |

GEE, Generalized estimating equations; PDA, patent ductus arteriosus; BPD, Bronchopulmonary Dysplasia; NEC, Necrotizing Enterocolitis

[a]Model 1: Adjusted ORs computed using multiple logistic regression models with GEE approach accounting for clustering within each site, and adjusted for: GA, sex, SGA, outborn, antenatal steroids, multiple births, respiratory severity score, pre-treatment PDA size

[b]Model 2: Inverse Probability Weighting Analysis estimated the predictive probability of the primary exposure based on GA, sex, SGA, outborn, antenatal steroids, multiple births, respiratory severity score , pre-treatment PDA size. Stabilization using marginal probability on weights was applied.

[C]With standard dose ibuprofen as reference, cells demonstrating statistically significant benefit are highlighted in green, statistically significant harm highlighted in red, no statistically significant difference highlighted in grey *(p value threshold of statistical significance <0.05)*

*The p-values were not adjusted for multiple comparisons*

© 2026 Mitra S et al. *JAMA Network Open.*

**eTable 6. Baseline demographic variables (per-protocol cohort)**

| Variables | Standard dose ibuprofen (n=285) | Adjustable dose ibuprofen (n=352) | Indomethacin (n=67) | Acetaminophen (n=85) | PDA not treated (n=259) |
|---|---|---|---|---|---|
| Gestational age at birth (weeks); Median, IQR | 25 (24, 27) | 25 (24, 26) | 26 (24, 27) | 25 (24, 26) | 26 (25, 27) |
| Gestational age <26 weeks at birth; N (%) | 150 (52.6) | 224 (62.9) | 29 (43.3) | 56 (65.9) | 90 (34.8) |
| Birth weight (grams); Mean (SD) | 842 (218) | 785 (222) | 831 (209) | 802 (226) | 938 (245) |
| Male sex; N (%) | 167 (58.6) | 195 (55.1) | 28 (41.8) | 49 (57.7) | 131 (50.6) |
| Small for gestational age (<10th percentile); N (%) | 26 (9.1) | 40 (11.4) | 13 (19.4) | 8 (9.4) | 17 (6.6) |
| Multiples; N (%) | 88 (30.9) | 92 (26.1) | 19 (28.4) | 27 (31.8) | 70 (27) |
| Outborn; N (%) | 35 (12.3) | 45 (12.8) | 13 (19.4) | 20 (23.5) | 46 (17.8) |
| Apgar score at 5 min; Median (IQR) | 6 (5, 7) | 7 (5, 8) | 7 (6, 8) | 6 (3, 8) | 7 (5, 9) |
| Number of patients who received >1 dose of surfactant, n (%) | 138 (48.4) | 157 (44.6) | 24 (35.8) | 31 (36.5) | 80 (30.9) |
| SNAPII score >20, n (%) | 106 (37.9) | 108 (30.7) | 25 (37.3) | 17 (20) | 62 (23.9) |
| Age at first treatment (days), median, IQR | 5 (3, 8) | 6 (4, 12) | 6 (4, 9) | 11 (7, 21) | NA |
| PDA size* (mm); Median, IQR | 2.1 (1.8, 2.5) | 2.0 (1.7, 2.4) | 2.0 (2.0, 2.5) | 2.3 (2.0, 2.7) | 1.7 (1.4, 2.1) |
| Respiratory severity score**; Median, IQR | 2.4 (1.8, 3.3) | 2.5 (2.0, 3.3) | 2.1 (1.5, 2.7) | 3.5 (2.5, 4.4) | 1.92 (1.5, 3.0) |

NA: Not applicable

*Estimated PDA size on (a) pre-treatment echocardiography for treated infants; or (b) first echocardiography for untreated infants

** Respiratory severity score assessed at time of (a) pre-treatment echocardiography for treated infants; or (b) first echocardiography for untreated infants

© 2026 Mitra S et al. *JAMA Network Open.*

**eTable 7. Crude rates of study outcomes in each treatment arm in the per-protocol cohort**

| Outcomes | Standard dose ibuprofen (N=285) | Adjustable dose ibuprofen (N=352) | Indomethacin (N=67) | Acetaminophen (N=85) |
|---|---|---|---|---|
| **Primary outcome, n (%)** | | | | |
| Failure of primary pharmacotherapy | 113 (39.7) | 161 (45.7) | 22 (32.8) | 42 (49.4) |
| **Secondary outcomes, n (%)** | | | | |
| Repeat medical therapy | 112 (39.3) | 160 (45.5) | 22 (32.8) | 40 (47.1) |
| Interventional PDA closure | 17 (6.0) | 37 (10.5) | 4 (6.0) | 4 (4.7) |
| Pre-discharge mortality | 40 (14.0) | 58 (16.5) | 2 (3.0) | 11 (14.0) |
| Moderate-Severe BPD | 177 (72.2) | 164 (46.6) | 55 (83.3) | 37 (49.3) |
| NEC (stage 2 or greater) | 33 (11.6) | 41 (11.7) | 6 (9.0) | 11 (12.9) |
| Definite sepsis following initiation of treatment | 77 (27.0) | 85 (24.2) | 1 (1.5) | 22 (25.9) |

© 2026 Mitra S et al. *JAMA Network Open.*

## eTable 8. Modeled estimates for study outcomes (per-protocol cohort)

| | **Model 1: Logistic regression model with GEE[a]** | | | **Model 2: Inverse Probability Weighted model[b]** | | |
|---|---|---|---|---|---|---|
| | **Adjustable dose ibuprofen** | **Indomethacin** | **Acetaminophen** | **Adjustable dose ibuprofen** | **Indomethacin** | **Acetaminophen** |
| **Outcomes** | ***Adjusted OR (95% CI) with standard dose ibuprofen as reference[c]*** | | | ***Adjusted OR (95% CI) with standard dose ibuprofen as reference[c]*** | | |
| **Failure of primary pharmacotherapy** | 1.19 (0.71, 2.00) | 0.7 (0.45, 1.08) | 1.34 (0.85, 2.12) | 1.21 (0.78, 1.88) | 1.22 (0.44, 3.35) | 1.85 (0.88, 3.9) |
| **Repeat medical therapy** | 1.21 (0.72, 2.04) | 0.72 (0.47, 1.09) | 1.29 (0.74, 2.23) | 1.22 (0.79, 1.90) | 1.24 (0.45, 3.41) | 1.87 (0.89, 3.93) |
| **Interventional PDA closure** | 2.1 (0.92, 4.78) | 1.09 (0.57, 2.07) | 0.43 (0.16, 1.15) | 2.13 (1.08, 4.20) | 0.98 (0.15, 6.51) | 0.7 (0.12, 4.03) |
| **Pre-discharge mortality** | 1.18 (0.95, 1.47) | 0.16 (0.14, 0.18) | 1.47 (1.12, 1.92) | 1.27 (0.76, 2.13) | 0.01 (<0.001, 0.28) | 1.9 (0.66, 5.44) |
| **Moderate-Severe BPD** | 0.39 (0.19, 0.81) | 2.06 (1.24, 3.44) | 0.25 (0.13, 0.48) | 0.35 (0.22, 0.55) | 2.92 (1.23, 6.90) | 0.25 (0.10, 0.59) |
| **NEC (stage 2 or greater)** | 0.98 (0.54, 1.79) | 0.95 (0.56, 1.60) | 1 (0.58, 1.74) | 0.92 (0.51, 1.66) | 1.96 (0.78, 4.88) | 1.3 (0.35, 4.85) |
| **Definite sepsis** | 0.93 (0.53, 1.65) | 0.07 (0.04, 0.10) | 1.68 (0.84, 3.34) | 0.95 (0.62, 1.45) | 0.03 (0.003, 0.29) | 2.44 (1.15, 5.16) |

GEE, Generalized estimating equations; PDA, patent ductus arteriosus; BPD, Bronchopulmonary Dysplasia; NEC, Necrotizing Enterocolitis

[a]Model 1: Adjusted ORs computed using multiple logistic regression models with GEE approach accounting for clustering within each site, and adjusted for: GA, sex, SGA, outborn, antenatal steroids, multiple births, respiratory severity score, pre-treatment PDA size

[b]Model 2: Inverse Probability Weighting analysis estimated the predictive probability of the primary exposure based on GA, sex, SGA, outborn, antenatal steroids, multiple births, respiratory severity score , pre-treatment PDA size. Stabilization using marginal probability on weights was applied.

[C]With standard dose ibuprofen as reference, cells demonstrating statistically significant benefit are highlighted in green, statistically significant harm highlighted in red, no statistically significant difference highlighted in grey *(p value threshold of statistical significance <0.05)*

*The p-values were not adjusted for multiple comparisons*

© 2026 Mitra S et al. *JAMA Network Open.*

**eTable 9. Subgroup analysis of the drug dose effectiveness cohort based on timing of treatment**

eTable 9a. Infants treated <7 days (n=633)

| **Outcomes** | **Adjustable dose ibuprofen (N=22)** | **Indomethacin (N=66)** | **Acetaminophen (N=147)** |
|---|---|---|---|
| | ***Adjusted OR (95% CI) with standard dose ibuprofen (n=398) as reference[a]*** | | |
| Failure of primary pharmacotherapy | 0.61 (0.17, 2.13) | 2.32 (1.07, 5.06) | 1.58 (0.96, 2.58) |
| Pre-discharge mortality | 0.95 (0.19, 4.67) | 0.16 (0.03, 0.96) | 1.12 (0.61, 2.06) |
| Moderate-Severe BPD | 0.81 (0.23, 2.87) | 2.69 (1.19, 6.11) | 0.79 (0.45, 1.40) |
| NEC (stage 2 or greater) | 1.43 (0.21, 9.74) | 2.16 (0.79, 5.89) | 0.95 (0.48, 1.88) |

eTable 9b. Infants treated ≥7 days (n=464)

| **Outcomes** | **Adjustable dose ibuprofen N=135** | **Indomethacin N=23** | **Acetaminophen N=170** |
|---|---|---|---|
| | ***Adjusted OR (95% CI) with standard dose ibuprofen (n=136) as reference[a]*** | | |
| Failure of primary pharmacotherapy | 0.68 (0.35, 1.31) | 0.05 (0.01, 0.31) | 0.36 (0.18, 0.70) |
| Pre-discharge mortality | 2.57 (0.88, 7.49) | <0.01 (<0.01, <0.01) | 2.17 (0.71, 6.63) |
| Moderate-Severe BPD | 0.34 (0.17, 0.66) | 7.67 (1.75, 33.74) | 0.54 (0.28, 1.08) |
| NEC (stage 2 or greater) | 2.19 (0.63, 7.61) | <0.01 (<0.01, <0.01) | 6.11 (1.96, 19.02) |

[a]With standard dose ibuprofen as reference, Inverse Probability Weighting Analysis estimated the predictive probability of the primary exposure based on GA, sex, SGA, outborn, antenatal steroids, multiple births, respiratory severity score, pre-treatment PDA size. Stabilization using marginal probability on weights was applied.

© 2026 Mitra S et al. *JAMA Network Open.*

## eTable 10. Subgroup analysis of the drug dose effectiveness cohort based on gestational age cut-off

eTable 10a. GA < 26 weeks (n=651)

| **Outcomes** | **Adjustable dose ibuprofen N=86** | **Indomethacin N=46** | **Acetaminophen N=195** |
|---|---|---|---|
| | ***Adjusted OR (95% CI) with standard dose ibuprofen (n=324) as reference[a]*** | | |
| Failure of primary pharmacotherapy | 0.91 (0.48, 1.73) | 0.66 (0.17, 2.57) | 1.09 (0.63, 1.91) |
| Pre-discharge mortality | 1.77 (0.82, 3.85) | 0.1 (0.01, 0.82) | 1.12 (0.59, 2.12) |
| Moderate-Severe BPD | 0.33 (0.14, 0.75) | 11.2 (2.93, 42.95) | 0.74 (0.40, 1.37) |
| NEC (stage 2 or greater) | 1.45 (0.63, 3.36) | 1.09 (0.28, 4.31) | 1.35 (0.76, 2.41) |

eTable 10b. GA ≥ 26 weeks (n=446)

| **Outcomes** | **Adjustable dose ibuprofen N=71** | **Indomethacin N=43** | **Acetaminophen N=122** |
|---|---|---|---|
| | ***Adjusted OR (95% CI) with standard dose ibuprofen (n=210) as reference[a]*** | | |
| Failure of primary pharmacotherapy | 2.36 (0.96, 5.79) | 0.47 (0.17, 1.26) | 1.18 (0.63, 2.22) |
| Pre-discharge mortality | 0.29 (0.03, 2.54) | 1.44 (0.25, 8.45) | 2. 49 (0.99, 6.26) |
| Moderate-Severe BPD | 0.38 (0.18, 0.82) | 2.07 (0.85, 5.05) | 0.54 (0.27, 1.1) |
| NEC (stage 2 or greater) | 2.42 (0.44, 13.34) | 1.77 (0.25, 12.63) | 2.35 (0.65, 8.58) |

[a]With standard dose ibuprofen as reference, Inverse Probability Weighting Analysis estimated the predictive probability of the primary exposure based on GA, sex, SGA, outborn, antenatal steroids, multiple births, respiratory severity score, pre-treatment PDA size. Stabilization using marginal probability on weights was applied.

© 2026 Mitra S et al. *JAMA Network Open.*

## eTable 11. Sensitivity Analysis of the GEE models adjusting for variance estimates (accounting for the small number of clusters)

eTable 11a: Sensitivity Analysis of the GEE model for the drug dose effectiveness cohort adjusting for variance estimates accounting for clustering within each site

| | **Model: Logistic regression model with GEE accounting for clustering within each site[a]** | | |
|---|---|---|---|
| | **Adjustable dose ibuprofen** | **Indomethacin** | **Acetaminophen** |
| **Outcomes** | ***Adjusted Odds Ratios (95% Confidence Intervals) with standard dose ibuprofen as reference*** | | |
| **Failure of primary pharmacotherapy** | 1.30 (0.74, 2.27) | 0.88 (0.07, 10.57) | 1.01 (0.57, 1.80) |
| **Repeat medical therapy** | 1.42 (0.73, 2.76) | 0.90 (0.07, 10.70) | 0.97 (0.48, 1.96) |
| **Interventional PDA closure** | 0.54 (0.25, 1.19) | 0.63 (0.14, 2.74) | 0.82 (0.37, 1.83) |
| **Pre-discharge mortality** | 1.48 (0.51, 4.25) | 0.32 (0.03, 3.06) | 1.38 (0.95, 1.99) |
| **Moderate-Severe BPD** | 0.54 (0.16, 1.78) | 2.56 (0.6, 24.88) | 0.68 (0.32, 1.44) |
| **NEC (stage 2 or greater)** | 1.07 (0.50, 2.26) | 0.80 (0.30, 2.16) | 1.39 (0.70, 2.75) |
| **Definite sepsis** | 1.08 (0.52, 2.24) | **0.21 (<0.01, 6.41)** | 1.00 (0.61, 1.65) |

GEE, Generalized estimating equations; PDA, patent ductus arteriosus; BPD, Bronchopulmonary Dysplasia; NEC, Necrotizing Enterocolitis

[a]***Model description:*** Adjusted ORs computed using multiple logistic regression models with GEE approach accounting for clustering within each site, and using a small sample corrected empirical variance estimator (Mancl and DeRouen correction), and adjusted for: GA, sex, SGA, outborn, antenatal steroids, multiple births, respiratory severity score, pre-treatment PDA size

© 2026 Mitra S et al. *JAMA Network Open.*

eTable 11b. Sensitivity Analysis of the GEE model for clinical outcomes of PDA treated vs conservatively managed infants adjusting for variance estimates accounting for clustering within each site

| **Clinical Outcomes** | **PDA treatment provided (n=1097)** | **PDA diagnosed but not treated (n=259)** | **Model 1: Logistic regression model with GEE[a] *[Adjusted odd ratios (95% CI)]*** |
| --- | --- | --- | --- |
| **Pre-discharge mortality, n (%)** | 159 (14.5%) | 40 (15.4%) | 0.60 (0.28, 1.30) |
| **Moderate-Severe BPD, n (%)** | 606 (55.2%) | 84 (32.4%) | **2.38 (1.06, 5.35)** |
| **NEC (stage 2 or greater), n (%)** | 127 (11.6%) | 16 (6.2%) | 1.50 (0.93, 2.40) |
| **Definite sepsis, n (%)** | 250 (22.8%) | 52 (21.2%) | 0.86 (0.59, 1.26) |

GEE, Generalized estimating equations; PDA, patent ductus arteriosus; BPD, Bronchopulmonary Dysplasia; NEC, Necrotizing Enterocolitis

[a]***Model description***: Adjusted ORs computed using multiple logistic regression models with GEE approach accounting for clustering within each site, and using a small sample corrected empirical variance estimator (Mancl and DeRouen correction), and adjusted for: GA, sex, SGA, outborn, antenatal steroids, multiple births, respiratory severity score, pre-treatment PDA size

© 2026 Mitra S et al. *JAMA Network Open.*
