# Supplementary material for: Pharmacologic Therapies for Patent Ductus Arteriosus in Extremely Preterm Infants
Source: JAMA Netw Open. 2026 Jun 9;9(6):e2617477. doi: 10.1001/jamanetworkopen.2026.17477 (PMC13250707; doi:10.1001/jamanetworkopen.2026.17477)
Supplement: Supplement 2. — Nonauthor Collaborators [file jamanetwopen-e2617477-s002.pdf]

Supplemental Online Content: Nonauthor Collaborators
*First name, last name, and suffix (if applicable) are required and will appear in PubMed.

***Group Name(s): Canadian Neonatal Network**

***First Name and Middle Initial(s)**	***Last Name**	***Suffix (eg, Jr, III)**	Academic Degrees	Institution	Location (city, state/province, country)	Role or Contribution, eg, chair, principal investigator	Group (if more than 1 Group listed in the byline) and/or Subgroup (eg, Steering Committee)
Marc	Beltempo		MD	Montreal Children's Hospital at McGill University Health Centre	Montréal, Québec, Canada	Director	
Marco	Zeid		NNP, MSc	Montreal Children's Hospital at McGill University Health Centre	Montréal, Québec, Canada	Site Investigator	
Prakesh S	Shah		MD, MSc	Mount Sinai Hospital	Toronto, Ontario, Canada	Site Investigator	
Thevanisha	Pillay		MD	Victoria General Hospital	Victoria, British Columbia, Canada	Site Investigator	
Jonathan	Wong		MD	British Columbia Women's Hospital	Vancouver, British Columbia, Canada	Site Investigator	
Miroslav	Stavel		MD	Royal Columbian Hospital	New Westminster, British Columbia, Canada	Site Investigator	
Rebecca	Sherlock		MD	Surrey Memorial Hospital	Surrey, British Columbia, Canada	Site Investigator	
Ayman A	Mehrem		MD	Foothills Medical Centre	Calgary, Alberta, Canada	Site Investigator	
Joseph	Ting		MD	Royal Alexandra Hospital and University of Alberta Hospital	Edmonton, Alberta, Canada	Site Investigator	
Carlos	Fajardo		MD	Alberta Children's Hospital	Calgary, Alberta, Canada	Site Investigator	
Andrei	Harabor		MD	Regina General Hospital	Regina, Saskatchewan, Canada	Site Investigator	
Jaya	Bodani		MD	Regina General Hospital	Regina, Saskatchewan, Canada	Former Site Investigator	
Lannae	Strueby		MD	Jim Pattison Children's Hospital	Saskatoon, Saskatchewan, Canada	Site Investigator	
Mary	Seshia		MBChB	Winnipeg Health Sciences Centre	Winnipeg, Manitoba, Canada	Site Investigator	

Supplemental Online Content: Nonauthor Collaborators
*First name, last name, and suffix (if applicable) are required and will appear in PubMed.

*First Name and Middle Initial(s)	*Last Name	*Suffix (eg, Jr, III)	Academic Degrees	Institution	Location (city, state/province, country)	Role or Contribution, eg, chair, principal investigator	Group (if more than 1 Group listed in the byline) and/or Subgroup (eg, Steering Committee)
Deepak	Louis		MD	Winnipeg Health Sciences Centre	Winnipeg, Manitoba, Canada	Site Investigator	
Chelsea	Ruth		MD	St. Boniface General Hospital	Winnipeg, Manitoba, Canada	Site Investigator	
Ann	Yi		MD	St. Boniface General Hospital	Winnipeg, Manitoba, Canada	Site Investigator	
Amit	Mukerji		MD	Hamilton Health Sciences Centre	Hamilton, Ontario, Canada	Site Investigator	
Kevin	Coughlin		MD	London Health Sciences Centre	London, Ontario, Canada	Site Investigator	
Sajit	Augustine		MD	Windsor Regional Hospital	Windsor, Ontario, Canada	Site Investigator	
Kyong-Soon	Lee		MD, MSc	Hospital for Sick Children	Toronto, Ontario, Canada	Site Investigator	
Eugene	Ng		MD	Sunnybrook Health Sciences Centre	Toronto, Ontario, Canada	Site Investigator	
Brigitte	Lemyre		MD	The Ottawa Hospital and Children's Hospital of Eastern Ontario	Ottawa, Ontario, Canada	Site Investigator	
Eyad	Bitar		MD	Kingston General Hospital	Kingston, Ontario, Canada	Site Investigator	
Victoria	Bizgu		MD	Jewish General Hospital	Montréal, Québec, Canada	Site Investigator	
Nina	Nouraeyan		MD	Jewish General Hospital	Montréal, Québec, Canada	Site Investigator	
Keith	Barrington		MBChB	Hôpital Sainte-Justine	Montréal, Québec, Canada	Site Investigator	
Anie	Lapointe		MD	Hôpital Sainte-Justine	Montréal, Québec, Canada	Site Investigator	
Guillaume	Ethier		NNP	Hôpital Sainte-Justine	Montréal, Québec, Canada	Site Investigator	

Supplemental Online Content: Nonauthor Collaborators
*First name, last name, and suffix (if applicable) are required and will appear in PubMed.

***First Name and Middle Initial(s)**	***Last Name**	***Suffix (eg, Jr, III)**	Academic Degrees	Institution	Location (city, state/province, country)	Role or Contribution, eg, chair, principal investigator	Group (if more than 1 Group listed in the byline) and/or Subgroup (eg, Steering Committee)
Marie	St-Hilaire		MD	Hôpital Maisonneuve-Rosemont	Montréal, Québec, Canada	Site Investigator	
Valerie	Bertelle		MD	Centre Hospitalier Universitaire de Sherbrooke	Sherbrooke, Québec, Canada	Site Investigator	
Edith	Masse		MD	Centre Hospitalier Universitaire de Sherbrooke	Sherbrooke, Québec, Canada	Site Investigator	
Paloma	Costa		MD	Moncton Hospital	Moncton, New Brunswick, Canada	Site Investigator	
Hala	Makary		MD	Dr. Everett Chalmers Hospital	Fredericton, New Brunswick, Canada	Site Investigator	
Ahmad	Aziz		MD	Dr. Everett Chalmers Hospital	Fredericton, New Brunswick, Canada	Site Investigator	
Gabriela de Carvalho	Nunes		MD	Saint John Regional Hospital	Saint John, New Brunswick, Canada	Site Investigator	
Wissam	Alburaki		MD	Saint John Regional Hospital	Saint John, New Brunswick, Canada	Site Investigator	
Jo-Anna	Hudson		MD	Janeway Children’s Health and Rehabilitation Centre	St. John’s, Newfoundland, Canada	Site Investigator	
Jehier	Afifi		MBChB, MSc	IWK Health Centre	Halifax, Nova Scotia, Canada	Site Investigator	
Andrzej	Kajetanowicz		MD	Cape Breton Regional Hospital	Sydney, Nova Scotia, Canada	Site Investigator	
Catherine	Chang		MD	University of Utah	Salt Lake City, Utah, United States of America	Site Investigator	
Christine	Drolet		MD	Centre Hospitalier Universitaire de Québec	Sainte Foy, Québec, Canada	Site Investigator	
Bruno	Piedboeuf		MD	Centre Hospitalier Universitaire de Québec	Sainte Foy, Québec, Canada	Site Investigator	
