# Supplementary material for: Pharmacologic Therapies for Patent Ductus Arteriosus in Extremely Preterm Infants
Source: JAMA Netw Open. 2026 Jun 9;9(6):e2617477. doi: 10.1001/jamanetworkopen.2026.17477 (PMC13250707; doi:10.1001/jamanetworkopen.2026.17477)
Supplement: Supplement 3. — Data Sharing Statement [file jamanetwopen-e2617477-s003.pdf]

Data Sharing Statement

Mitra. Pharmacologic Therapies for Patent Ductus Arteriosus in Extremely Preterm Infants.
JAMA Netw Open. Published June 09, 2026. doi:10.1001/jamanetworkopen.2026.17477

Data
Data available: Yes
Data types: Deidentified participant data
How to access data: souvik.mitra@cw.bc.ca
When available: With publication

Supporting Documents
Document types: None

Additional Information
Who can access the data: researchers whose proposed use of the data has been approved
Types of analyses: for any inferential statistical analysis
Mechanisms of data availability: with a signed data access agreement
